# Sex differences in anatomic plasticity of gut neuronal–mast cell interactions

**DOI:** 10.14814/phy2.15066

**Published:** 2021-10-04

**Authors:** Luke A. Schwerdtfeger, Stuart A. Tobet

**Affiliations:** ^1^ Department of Biomedical Sciences Colorado State University Fort Collins Colorado USA; ^2^ School of Biomedical Engineering Colorado State University Fort Collins Colorado USA

**Keywords:** gut, intestine, mast cell, mucosa, VIP

## Abstract

The gut wall houses mast cells that are anatomically situated near enteric neuronal fibers. Roles of specific neuropeptides in modulating function of immune components like mast cells in response to challenge with bacterial components are relatively unknown. Investigating such interactions requires models that include diverse cellular elements in native anatomic arrangements. Using an organotypic slice model that maintains gut wall cellular diversity ex vivo, the present study compared responses between tissues derived from male and female mice to examine neural‐immune signaling in the gut wall after selected treatments. Ileum slices were treated with pharmacological reagents that block neuronal function (e.g., tetrodotoxin) or vasoactive intestinal peptide (VIP) receptors prior to challenge with lipopolysaccharide (LPS) to assess their influence on anatomic plasticity of VIP fibers and activation of mast cells. Sex differences were observed in the number of mucosal mast cells (c‐kit/ACK2 immunoreactive) at baseline, regardless of treatment, with female ileum tissue having 46% more ACK2‐IR mast cells than males. After challenge with LPS, male mast cell counts rose to female levels. Furthermore, sex differences were observed in the percentage of ACK2‐IR cells within 1 µm of a VIP+ neuronal fiber, and mast cell size, a metric previously tied to activation, with females having larger cells at baseline. Male mast cell sizes reached female levels after LPS challenge. This study suggests sex differences in neural‐immune plasticity and in mast cell activation both basally and in response to challenge with LPS. These sex differences could potentially impact functional neuroimmune response to pathogens.

## Graphical abstract

Gut wall mast cells have sex differences in size, quantity, and interactions with VIP neuronal fibers. Response of mast cells to challenge with lipopolysaccharide was sexually different. These sex differences could be important in how the host gut responds to pathogen.

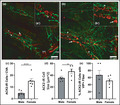

## 1 INTRODUCTION

Neural regulation of immune components throughout the gut wall has gained increased attention. Investigating complex neural‐immune signaling events in the gut requires the use of culture systems that maintain diverse cellular elements in their natural arrangements. Mast cells are a multifunctional population of immune cells anatomically situated near myriad enteric neuronal fibers in the gut mucosa (Stead et al., 1989). Neural stimuli have been shown to alter mast cell function (Jacobson et al., 2021; Piotrowski & Foreman, 1985). Mucosal immune cells, like mast cells among others, express neuropeptide receptors for vasoactive intestinal peptide (VIP; Keita et al., 2013), calcitonin gene‐related peptide (CGRP), substance P, and others (Forsythe & Bienenstock, 2012). Roles of specific enteric neuronal populations in regulating bi‐directional communication with heterogenous mast cell subsets are important for understanding the neuroimmune axes of the gut.

VIP and pituitary adenylate cyclase‐activating polypeptide (PACAP) are vasoactive peptides transcribed and translated from different genes, but with significant sequence homology (Sikora et al., 1984) and are secreted by enteric neurons (Furness & Costa, 1979) and mucosal mast cells (Cutz et al., 1978). More than half of submucosal enteric neuronal fibers projecting throughout the mucosa of the small intestine are VIP‐immunoreactive (VIP‐IR; Mongardi Fantaguzzi et al., 2009). VIP‐IR neuronal fibers have been shown in close proximity to mast cells more regularly in rats with inflammatory bowel disease (Casado‐Bedmar et al., 2019). VIP signaling has also been shown to regulate gut smooth muscle contraction (Katsoulis et al., 1993) and enteric goblet cell production (Schwerdtfeger & Tobet, 2020) among other functions. VIP and PACAP bind VPAC receptors 1 and 2 at slightly different affinities (Vaudry et al., 2009) and both are immunoreactive to most antisera targeting VIP. Therefore, “VIP” shall refer to VIP and PACAP immunoreactivity and activity throughout this paper. VPAC receptors can be blocked using a receptor antagonist ([D‐p‐Cl‐ Phe^6^, Leu^17^]‐VIP; Pandol et al., 1986), and will be referred to as “VPACa.”

Mucosal mast cells are bone marrow progenitor‐derived immune cells that play roles in the gut ranging from inflammatory events via histamine release, to innate immune responses to pathogenic bacteria (Albert‐Bayo et al., 2019). Mucosal mast cells mature from mast cell precursors resident in blood and the gut wall, both of which express c‐kit/CD117 (Liu et al., 2010) which can be labeled immunohistochemically with an anti‐CD117 antibody (ACK2). Mucosal mast cells have been shown to sense and respond to bacteria and their wall components like lipopolysaccharide (LPS) during barrier breach of the gut wall (Malaviya et al., 1996; Piliponsky & Romani, 2018). Mast cells are also capable of producing and releasing VIP in response to LPS stimulation (Martinez et al., 1999). Neural regulation of the mast cell responses to pathogen challenge is largely unknown, partially due to the requirement of culture methods that maintain all three cellular components in proper anatomic arrangements.

Several immunological sex differences have been reported, with females tending toward more adaptive immune cell expression and basal levels of inflammation in healthy states (Klein & Flanagan, 2016). Females are more likely to have gut pathologies such as irritable bowel syndrome (Lovell & Ford, 2012), and mucosal mast cell counts are increased in certain pathologies in females compared to males (Cremon et al., 2009; Lee et al., 2016). However, the presence of basal sex differences in immune components, including mast cells, has not been well studied in intestinal cell populations. Neuronal modulation of mast cell production and secretion pathways is an unexplored target for better understanding of the effect sex plays in neuroimmune communications in the gut wall in healthy and perturbed states.

This study harnesses an organotypic intestinal slice model that has previously been shown to maintain slices of mouse intestine with neural, immune, epithelial, and bacterial components ex vivo (Schwerdtfeger et al., 2016), and can be challenged with bacterial antigens like LPS (Schwerdtfeger & Tobet, 2020). Organotypic gut slices were recently used to show the dense apposition of goblet cells by neuronal fibers in the mouse ileum, with VIP antagonism inhibiting production of new goblet cells (Schwerdtfeger & Tobet, 2020). These results pointed toward the importance of VIP in gut epithelial functional regulation. The immune system is a critical component of gut wall function and was investigated in the context of VIP neuronal regulation of mast cell activation and function with respect to sex, and post‐challenge with LPS.

## 2 MATERIALS AND METHODS

### 2.1 Animals

Mice between 8 and 12 weeks of age and both sexes were used for all experiments. Animals were housed at Colorado State University under the care of Laboratory Animal Resources. Mice were housed in cages with aspen bedding (autoclaved Sani‐chips; Harlan Teklad, Madison, WI) with ad libitum access to food (No. 8649; Harlan Teklad) and water under a 14:10‐h light–dark cycle. All slices were generated from mice (C57BL/6 background) expressing yellow fluorescent protein (YFP) driven by a neuronally selective promoter (Thy‐1) as previously described (Thy‐1 YFP; Feng et al., 2000). Animal studies were approved by the Colorado State University IACUC under protocol #1021.

### 2.2 Organotypic slice preparation

Preparation of intestinal slices has been previously described at length (Schwerdtfeger et al., 2016; Schwerdtfeger & Tobet, 2020), and was performed similarly in the current study. Briefly, mice were deeply anesthetized with isoflurane and killed via decapitation. The small intestine was removed from the pylorus–duodenal junction to the ileocecal junction. Tissue was immediately placed into 4°C 1X Krebs buffer (in mM: 126 NaCl, 2.5 KCl, 2.5 CaCl_2_, 1.2 NaH_2_PO_4_, and 1.2 MgCl_2_) and the ileum was separated and cut into pieces roughly 2–4 mm in length. Tissue was blocked in 8% low melting point agarose (Gold Biotechnology) until polymerization prior to cutting on a vibrating microtome (VT1000S; Leica Microsystems) at 250 µm thick. Slices were transferred into a 60‐mm plastic bottom dish (Corning Inc) containing 5 ml of Hibernate Media (Life Technologies). Slices spent 15 min at 4°C in hibernate media prior to removal of hibernate and addition of 5 ml of CTS Neurobasal‐A Media (ANB; Life Technologies) with 5% B‐27 supplement (B‐27; Life Technologies) where they spent 35 min at 37°C. Samples were plated on 35‐mm plastic bottom dishes (MatTek) and covered by a thin layer of collagen solution [vol/vol: 10.4% 10X MEM (Minimal Essential Medium, Sigma‐Aldrich), 4.2% sodium bicarbonate, and 83.5% collagen (PureCol; Inamed)] until polymerized before a final addition of 1 ml of ANB + B‐27. Tissue was left in at 37°C, 5% CO_2_, and 1% O_2_ incubator until experiments were performed.

### 2.3 Drug treatments

Slices of mouse ileum were treated in accordance with Table 1. Doses were chosen per previous experiments and preliminary dose–response analyses where physiologic effects on slices were seen within the first 24 h of culture (Schwerdtfeger et al., 2016; Schwerdtfeger & Tobet, 2020), slices were treated at 24 h ex vivo for a subsequent 24 h. To test potentially protective effects of treatments, drugs were added to slice dishes prophylactically at 0 h ex vivo prior to addition of LPS at 24 h ex vivo for 4 h. Slices were fixed in 4% formaldehyde for 15 min and subsequently washed 3x in phosphate‐buffered saline.

**TABLE 1:** Drug treatments used throughout experiments

Treatment	Concentration	Source
Vasoactive Intestinal Peptide (VIP)	10 µM	ABBIOTEC (Escondido, CA)
[D‐p‐Cl‐Phe6,eu17]‐VIP (VPACa)	10 µM	Bio‐Techne corporation (Minneapolis, MN)
Tetrodotoxin (TTX)	10 µM	Abcam (Cambridge, MA)
TLR‐grade Lipopolysaccharide (LPS)	10 µg/ml	Enzo Life Sciences, Inc. (Farmingdale, NY)

### 2.4 Re‐sectioning of slices

To improve visualization of cellular elements, post‐fixation, 250 µm slices were re‐sectioned at 50 µm as previously described (Schwerdtfeger & Tobet, 2020). Briefly, slices were embedded in 4% agarose solution (w/v; Fisher Scientific) and subsequently put in a 4°C fridge for 4 min to allow for agarose polymerization. Ileum slices were then re‐sectioned on a vibrating microtome (VT1000S; Leica Microsystems) at 50 µm thick prior to processing for immunohistochemistry.

### 2.5 Immunohistochemistry

Immunohistochemistry (IHC) was performed as previously described (Schwerdtfeger et al., 2016; Schwerdtfeger & Tobet, 2020). Briefly, 50 µm re‐sectioned tissue sections received 0.1 M glycine made in 0.05 M PBS for 30 min prior to three PBS washes before 15 min in 0.5% sodium borohydride in PBS. Sections were subsequently washed two times in PBS prior to blocking for 30 min in 5% normal goat serum (NGS; Fisher Scientific), 0.5% Tx, and 1% H_2_O_2_ in PBS. Sections then received 2.0 µg/ml of anti‐c‐kit 2 (ACK2; Novus Biologicals, Centennial, CO; CAT# NBP1‐433359) primary antibody in PBS with 1% BSA and 0.3% Tx. After incubation in the first primary, tissue sections were washed with 1% NGS in PBS four times for a total of 1 h. Next, a biotinylated donkey anti‐rat secondary (Jackson ImmunoResearch Inc, CAT# 712066150) made with 1% NGS and 0.5% Tx in PBS was added for 2 h at room temperature. Secondary antibody was washed out with four 15 min washes composed of 0.02% Tx in PBS. Sections were next incubated with Alexa Fluor 555–streptavidin (Invitrogen, CAT# S21381) conjugated tertiary with solution composed of 0.32% Tx in PBS for 1 h. Sections then received three PBS washes prior to the addition of the secondary primary antisera composed of anti‐VIP (Immunostar, Inc., CAT# 20077) at 1:8000 dilution in PBS with 1% BSA and 0.3% Tx. Post‐primary incubation, tissue sections were washed four times for a total of 1 h in PBS with 1% NGS prior to the addition of an Alexa Fluor 488‐conjugated goat anti‐rabbit secondary (Invitrogen, CAT# A11008) for 1 h. After secondary antibody, sections were washed three times in PBS before mounting on slides with Aqua‐Poly/Mount (Polysciences, Inc.) and subsequent imaging. All IHC experiments were performed with negative controls, where primary antibody was omitted, and pertinent detection antibodies were added.

### 2.6 Tissue imaging and analysis

Randomly selected ex vivo slices were imaged on a Nikon TE2000‐U inverted microscope using a 10X Plan‐Fluor objective and a UniBlitz shutter system (incent Associates, Rochester, NY) to visualize Thy‐1 YFP fluorescence to confirm the presence of fluorescent neurons and neuronal fibers. This has been used as a rudimentary marker for tissue health, where the presence of neurons and anatomically arranged fluorescent fibers were used as measures (Schwerdtfeger et al., 2016). Fixation in 4% formaldehyde removed Thy‐1 YFP fluorescence in mucosal––projecting neuronal fibers, allowing visualization of VIP by IHC for green fluorescent probes. Baseline quantities of mast cells and VIP neuronal fiber interactions were performed on cardiac perfused ileum tissues.

To assess mast cells and VIP in vivo, cardiac perfusions were performed using 10 ml of heparinized saline at 5 ml/min perfusion rate using a syringe pump prior to 10 ml of 4% formaldehyde perfused the same. Post‐perfusion, ileum tissue was sectioned at 50 µm and re‐sectioned slices were imaged after mounting on a Zeiss LSM 880 confocal microscope with an Axiocam 503 mono camera (Carl Zeiss, Inc.). Z‐stacks were acquired in 1 µm intervals through all 50 µm of each tissue section using standard laser and excitation/emission settings for the specific fluorophores used in IHC.

All image analyses were performed using Fiji (v1.0; NIH). Max intensity Z‐projections were performed through the center 30 µm of each image stack. Regions of interest (ROI) around all clearly discernable ACK2‐IR cells were manually drawn by a researcher blinded to treatment. ACK2‐IR cell quantities were counted manually after performing one erosion in Fiji to remove spurious signal. VIP‐IR neuronal fiber appositions with ACK2‐IR cells were counted by performing a 1 µm dilation on ACK2‐IR cell ROIs which provided a global 1 µm increased diameter on the cells. Image on image subtractions were performed to quantify where and how regularly ACK2‐IR cells had a VIP‐IR fiber within their enlarged diameter.

### 2.7 Statistics

**FIGURE 1:**
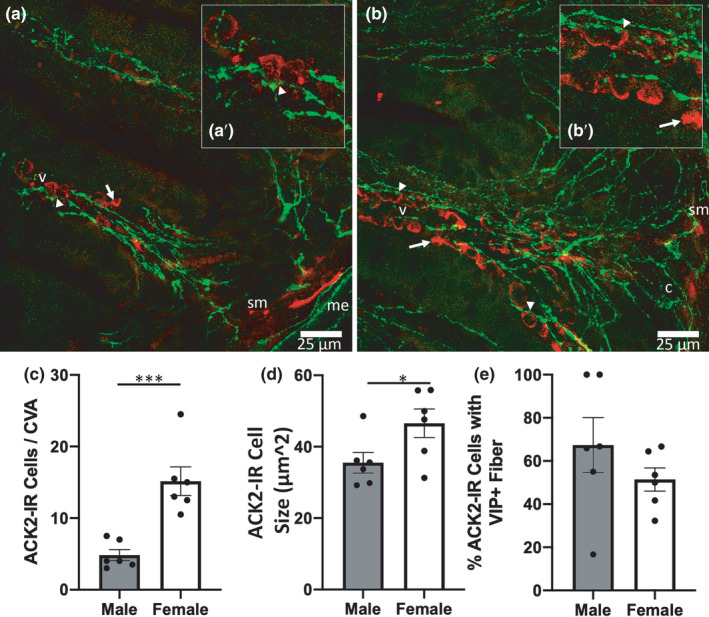
Basal sex difference in mast cell count per crypt‐villus axis (CVA) and mean ACK2 cell size were observed. Representative images from male (a) and female (b) ileum samples with arrows denoting representative ACK2 immunoreactive (IR) cells (red) not near a VIP‐immunoreactive fiber (green), while arrow heads point to exemplary ACK2‐IR cells within 1 μm of a VIP‐immunoreactive fiber. Cropped images in (a’) and (b’) show zoomed views of ACK2 cell proximity with VIP neuronal fibers. (c) quantification of ACK2 cell counts per CVA. Quantification of ACK2 cell size (d) and the percentage of ACK2‐IR cells within 1 μm of a VIP‐IR fiber (e). n = 6 males, 6 females. ‘sm’ denotes submucosa, ‘c’ crypt, and ‘me’ muscularis externa. Scale bars in (a) and (b) are both 25 μm

**FIGURE 2:**
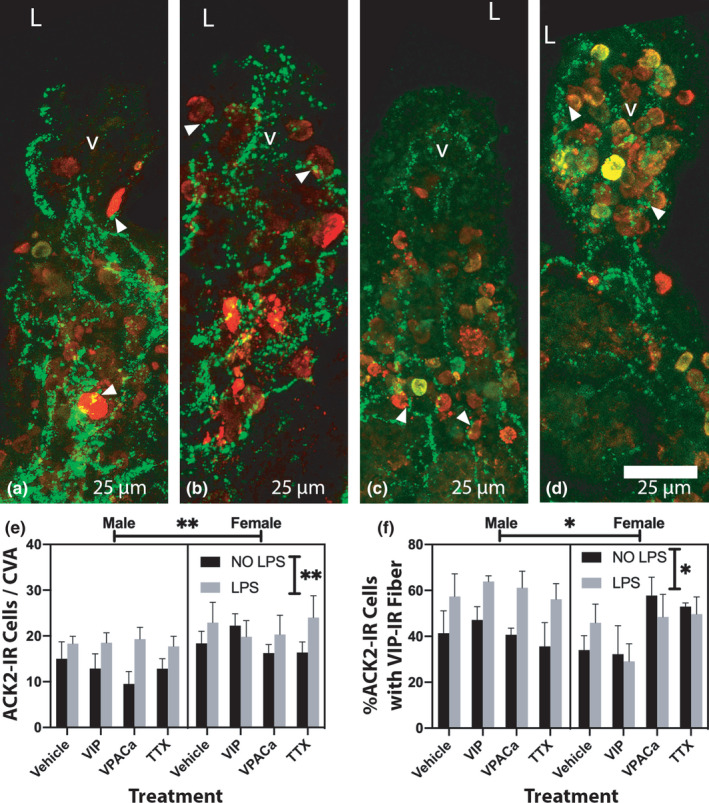
Sex and LPS effects were observed in ACK2‐IR cell counts per CVA regardless of treatment, and in the percentage of ACK2‐IR cells within 1 μm of a fiber. (a‐d) representative images from a male ileum treated with vehicle (a) and vehicle +LPS (B) showing ACK2‐IR cells (red) and VIP‐IR fibers (green). Representative images from female ileum treated with vehicle (c) or vehicle +LPS (d). (e) is a 3‐way ANOVA quantitation showing effects of sex and LPS treatment on ACK2 cell counts. (f) is a 3‐way ANOVA quantitation showing effects of sex and LPS treatment on the percentage of ACK2‐IR cells within 1 μm of a VIP‐IR fiber. Vertical significance bars in (e) and (f) denote an observed effect of LPS while horizontal significance bars denote observed sex effects. n = 4 male, 4 female in ‘NO LPS’ groups, and n = 5 male, 5 female in ‘LPS’ groups Arrow heads in (a‐d) point towards exemplary ACK2‐IR cells within 1 μm of a VIP‐IR fiber. ‘v’ denotes a villus, ‘L’ lumen. Scale bars are 25 μm in (a‐d). ‘*’ denotes a *p* < 0.05, and ‘**’ a *p* < 0.01

**FIGURE 3:**
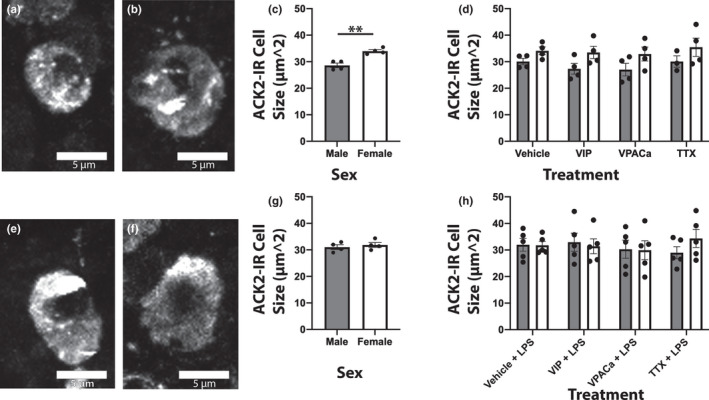
Female ACK2‐IR cells were larger than males, an effect lost when slices were challenged with LPS. (a/e) and (b/f) are representative images of ACK2‐immunoreactive cells from male and female ileum slices treated with vehicle (a/b) or vehicle + LPS (E/F) respectively. (c) and (d) are quantification of mean ACK2‐ IR cell size across treatments. (g) and (h) are quantification of mean ACK2‐IR cell size in slices challenged with LPS ex vivo. n = 4 male, 4 female in ‘NO LPS’ groups, and n = 5 male, 5 female in ‘LPS’ groups Scale bars in (a/e) and (b/f) are all 5 μm

**FIGURE 4:**
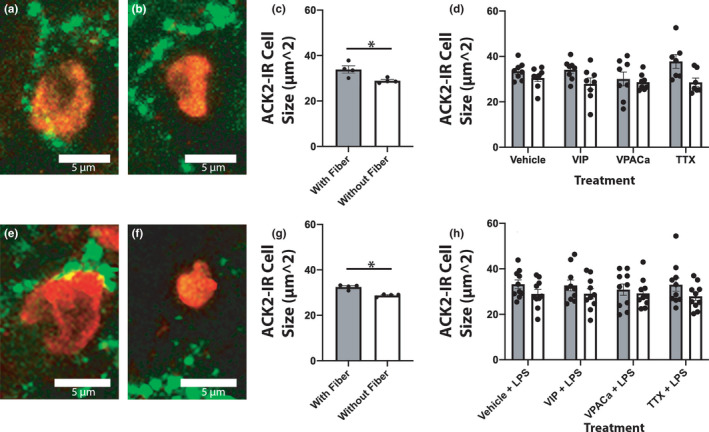
ACK2‐IR cells within 1 μm of a VIP‐IR neuronal fiber were larger than those not within 1 μm of a VIP‐IR fiber, regardless of treatment or challenge with LPS. (a/e) and (b/f) are representative images of ACK2‐immunoreactive cells (red) and VIP‐immunoreactive fibers (green) from a cell <1 μm from a VIP fiber or >1 μm from a fiber, respectively. Quantification in (c) and (d) of the mean ACK2 cell size in μm2 by treatment. (g) and (h) are quantification in the same fashion as (c/d) but in slices challenged with LPS. n = 8 animals Scale bars in (a/e) and (b/f) are all 5 μm

**FIGURE 5:**
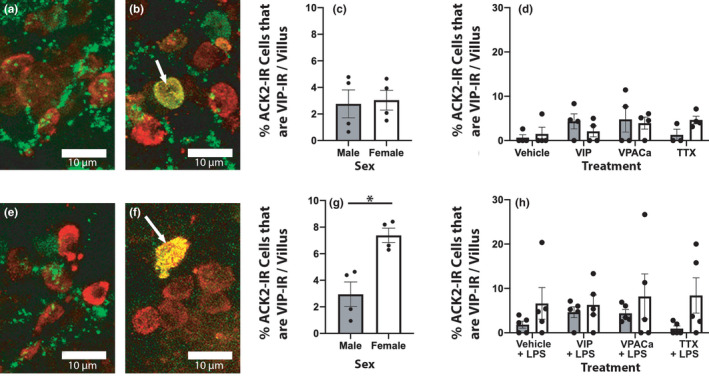
Slices from female ileums had more ACK2‐IR cells also immunoreactive to VIP in response to LPS challenge than males. (a/e) and (b/f) are representative images of ACK2 immunoreactivity (red) and VIP immunoreactivity (green) with arrows pointing towards exemplary cells immunoreactive to both ACK2 and VIP. (c) and (d) are quantifications of the percentage of ACK2‐IR cells that were greater than 20% of their area immunoreactive for VIP. (g) and (h) are quantifications the same as in (c/d) but in slices challenged with LPS. n = 4 male, 4 female in ‘NO LPS’ groups, and n = 5 male, 5 female in ‘LPS’ groups Scale bars in (a/e) and (b/f) are all 10 μm

Statistical analyses were performed in Prism 9 (GraphPad). Comparisons of ACK2‐IR cell counts and the percentage of ACK2‐IR cells with a VIP‐IR fiber in Figure 1 were both performed using one‐way ANOVA. Data in Figure 2e, which looked at ACK2‐IR cell counts across sex, treatment, and LPS challenge, are analyzed via three‐way ANOVA with a post hoc Tukey's multiple comparison test to compare individual means. Data in Figure 2f also used a three‐way ANOVA with a Tukey's post hoc test. Data in Figures 3, 4, and 5 are compared using a two‐way ANOVA by sex and treatments. All data are presented as means +/‐ standard error of the mean (SEM).

Animal numbers are reported in figure legends, where n = values correspond to individual animals used for each experiment. Slices were generated from ileums of each animal then cultured two slices per dish as technical replicates, prior to addition of dish‐specific drug treatments. Quantification was performed on each technical replicate and averaged prior to analyses at an “animal” level.

## 3 RESULTS

In fixative perfused ileum sections, mucosal ACK2‐IR cells, presumed to be mast cells, were regularly opposed by VIP‐IR neuronal fibers in mouse distal ileum. Tissue from females had significantly more mast cells per crypt–villus axis (CVA) than male Figure 1a,b/a',b') (Figure 1c; *F*(1,10) = 23.0, *p* < 0.01). ACK2‐IR cells from female ileum sections were also significantly larger than males (Figure 1d; *F*(1,10) = 5.1, *p* < 0.05). Immunoreactivity to ACK2 was used to localize mast cells, and dual labeling with VIP showed large amounts of fiber appositions on ACK2‐IR mast cells throughout the lamina propria of male (Figure 1a/a') and female (Figure 1b/b') ileal tissue. There were no notable differences in the percentage of ACK2‐IR cells within 1 µm of a VIP‐IR neuronal fiber in perfused ileum sections (Figure 1e).

No sex difference in percentage area labeled with VIP‐IR or VIP intensity was noted. Over the entire CVA, males had 0.71 ± 0.22% area labeled with VIP‐IR while females had 0.90 ± 0.24% (*F*(1,10) = 0.57 *p* > 0.5). In addition, no effect of treatment (*F*(3,60) = 1.2 *p* > 0.2) or region of CVA (crypt, basal villus, apical villus) was observed as measured by two‐way ANOVA (*F*(2,60) = 1.5 *p* > 0.2). There was also no interaction observed between treatment or region in percentage of VIP‐IR area labeled (*F*(6,60) = 0.1 *p* > 0.9).

Sex and LPS effects were observed in ACK2‐IR cell counts per CVA regardless of treatment with neuronal regulators. Representative images show male (Figure 2a/b) and female (Figure 2c/d) ileal villi with immunofluorescent ACK2 (red) and immunofluorescent VIP (green) closely localized. Quantification of image sets from slices cultured ex vivo showed that treatment with vehicle, VIP, VPACa, or TTX, did not impact ACK2‐IR cell counts per CVA (*F*(3,55) = 0.4 *p* > 0.4), however, sex (*F*(1,55) = 7.8 *p* < 0.01) and LPS challenge (*F*(1,55) = 8.2 *p* < 0.01) both significantly influenced the ACK2‐IR cell counts per CVA. Female tissues had more ACK2‐IR cells than males, and LPS treatment increased ACK2‐IR counts, regardless of sex, as there was no interaction between sex and LPS challenge (Figure 2e; *F*(1,55) = 0.5 *p* > 0.4). In addition, the percentage of ACK2‐IR cells within 1 µm of a VIP‐IR fiber was not significantly different between sexes (*F*(1,55) = 2.8 *p* = 0.10), but was altered by challenge with LPS (*F*(1,55) = 4.8 *p* < 0.05). There was an interaction between sex and LPS challenge (*F*(1,55) = 5.9 *p* = 0.018) and between sex and treatment (*F*(3,55) = 2.9 *p* < 0.05). This effect was partially driven by VIP treatment in females, which had the lowest levels of % ACK2‐IR within 1 µm of a VIP‐IR fiber regardless of LPS challenge (Figure 2f).

Mast cell size was sex dependent, with ACK2‐IR cell sizes larger in females than in males. ACK2 area of immunoreactivity (µm^2^) was smaller in male slices (Figure 3a) than it was in female (Figure 3b), regardless of treatment (Figure 3c‐d; *F*(1,23) = 10 *p* < 0.01). When challenged with LPS, ACK2‐IR cell sizes from male mice (Figure 3e) were similar to those in females (Figure 3f), independent of treatment (Figure 3g‐h; *F*(1,32) = 0.1 *p* > 0.50). ACK2‐IR cells within 1 µm of a VIP‐IR neuronal fiber (Figure 4a/e) were larger than those not within 1 µm of a VIP fiber (Figure 4b/f), regardless of treatment (Figure 4c/d; *F*(1,54) = 10.8 *p* < 0.01). When challenged with LPS, those ACK2‐IR cells within 1 µm of a VIP‐IR fiber were still larger than those not near a fiber, regardless of treatment (Figure 4g/h; *F*(1,72) = 5.43 *p* < 0.05; for all groups in Figure 4).

There was a sex difference in the number of ACK2‐IR cells that were also heavily immunoreactive for VIP, but only in response to LPS challenge. Ileal tissue from males (Figure 5a/e) had an equivalent percentage of ACK2 cells also reactive for VIP per villus as females (Figure 5b/f), regardless of treatment (Figure 5c/d; *F*(1,23) = 0.06 *p* > 0.40). When challenged with LPS, the percentage of ACK2‐IR cells heavily immunoreactive for VIP in male tissue remained largely unchanged, while females increased significantly, regardless of treatment (Figure 5g/h; *F*(1,32) = 5.03 *p* < 0.05).

## 4 DISCUSSION

Complex and diverse cellular elements in the intestinal wall must work in concert to maintain a healthy epithelial and mucus barrier. Barrier systems work to prevent pathogen invasion, and immunologically fight foreign antigens and pathogens if they breach the barrier. The extent to which enteric neuronal peptides are determinant, or modulatory players among gut wall signaling circuits, is not well understood. Intestinal mucosal mast cells have been known to sit in close anatomic proximity to enteric neuronal fibers (Stead et al., 1989) and are capable of themselves producing a host of classical “neuro” peptides like VIP (Cutz et al., 1978). Mucosal mast cells also express VIP receptors (Keita et al., 2013), and produce VIP once exposed to LPS (Martinez et al., 1999). Data in this study document a sex difference in anatomic arrangement, cell size, and VIP production capacity of enteric mast cells. Many of the observed sex differences were eliminated upon challenge with LPS, potentially indicating differences in basal compared to activated immunological states of the gut mucosa of males and females. It is important to note that the ACK2‐IR cells observed in this study are likely not exclusively mast cells. While c‐kit/ACK2 is expressed in high quantities on mucosal mast cells, other cell types can also express c‐kit/ACK2. Further parsing out of mucosal c‐kit/ACK2 reactive cell populations and their interactions with enteric neuronal fibers is needed. Together, these data highlight the need to consider sex as a relevant variable when investigating enteric mast cell functional interactions with the rest of the gut wall.

Sex differences in immune components are relatively common, with female immune systems often being thought of as more “active” than those in males (Klein & Flanagan, 2016). Additionally, differential levels of mast cell quantities and activation states during various gut pathologies across sexes have previously been shown (Cremon et al., 2009; Lee et al., 2016). In the current study, there was a sex difference in mast cell counts (ACK2‐IR cells) at baseline in perfused ileum tissues, with females having higher cell counts. In ex vivo cultured slices, mast cell counts were higher at baseline in samples from female mice. Challenging with LPS reduced the observed sex difference in mast cell quantities, with males “catching up” to female levels. The observed sex‐dependent response to challenge with LPS points toward a cohort of mast cells in female ileums that are either less likely to respond to a bacterial antigen or are already at capacity in their quantities in the gut wall. Conversely, male mast cells increased their quantities within 24 h, pointing toward a potentially more active mast cell population in male ileums. Previous work has demonstrated immune cell proliferation in intestinal lamina propria using organotypic cultures of human colon (Schwerdtfeger et al., 2019), as measured by incorporation of the thymidine analog 5‐ethynyl‐2’‐deoxyuridine (EdU). Using EdU to probe proliferative capacity of ACK2‐IR cells in this study, preliminary data showed that three slices of ileum from one male (2.67 ± 0.67 EdU+ACK2+ cells/crypt) had more EdU+mast cells in response to challenge with LPS in their crypts than three slices of female ileum (0.67 ± 0.67 cells/crypt). This points toward the observed increase in male mast cell counts being potentially driven by proliferation at a local level, as these slices are removed from the body during culture and there is no external lymph node supply to shuttle lymphocytes. Further investigation into the source of increased ACK2‐IR cell counts observed post‐treatment is warranted, but beyond the scope of the current study.

Sex differences in mast cell sizes are not commonly reported, however, mast cell size has been correlated with activation, and secretory capacity, with mast cells being activated in vitro being larger in size (Levi‐Shaffer et al., 2000). Larger mast cells could provide increased cytoplasmic area for packaging and storing secretory granules. This principle is in‐line with a larger, potentially more “active” female mast cell at basal state observed in this study, compared to a smaller, less “active” male mast cell. When challenged with LPS, female mast cells did not change size, however, male mast cells increased in size compared to non‐LPS dosed slices and became equivalent with female mast cell sizes. This points toward a potentially basal state activation of female mast cells in murine ileum, a sex difference in mast cell activation state only rectified in males when bacterial antigens like LPS are presented to mast cells.

Measurements of anatomic plasticity can be useful indicators of potential cell–cell signaling pathways like neuronal‐immune networks. Proximity of mucosal mast cells to neuronal fibers has been tied to visceral pain levels in human irritable bowel syndrome patients (Barbara et al., 2004). This study also points toward mast cells in close proximity to neuronal fibers being more activated than those not near a fiber. In the present study, those mast cells that were within 1 µm of a VIP neuronal fiber were larger than those 1 µm of further away from a fiber, regardless of treatment. These data suggest a subset of more activated mast cells in close proximity to VIP neuronal fibers, echoing the human data from Barbara et al., 2004, while extending the hypothesis by specifying one specific neuronal fiber type potentially involved in activated mast cell interactions with neuronal fibers.

Mucosal mast cells are capable of producing numerous classical neuropeptides, including VIP, however, sex differences in this peptide production have not been shown. While baseline levels of VIP production have been observed in mast cells, the quantities are relatively minimal (Cutz et al., 1978) and we did not observe VIP‐IR mast cells in perfusion fixed ileum sections in the present work. Challenging mast cells with LPS was shown previously to trigger production and subsequent release of VIP (Martinez et al., 1999). In this study, we did not observe sex differences, irrespective of treatment, in the percentage of mast cells “producing” VIP as indicated by VIP‐IR in ex vivo slices. However, when challenged with LPS, there was an increase in the percentage of VIP‐IR mast cells in female tissues only. Given that a small number of VIP‐IR cell bodies were observed in close proximity to ACK2‐IR cells (Figure 5), there is potential that other immune elements producing VIP are involved in these pathways. This sex difference indicates that female mucosal mast cells are potentially more readily able to produce VIP in response to bacterial antigens like LPS, however, the downstream function of mast cell produced VIP after LPS challenge needs further investigation.

In conclusion, the results of this study illuminate a sex difference in the anatomical localization and plasticity of neuronal–mast cell interactions, and in mast cell size and capacity to produce the neuropeptide VIP. Female neuro‐immune signaling seems to be in a more activated basal state, however, dosing with VIP or its antagonists did not substantially alter female or male mast cell numbers or size or VIP–mast cell anatomic relations or interactions. Not until LPS challenge did males recover from their decreased mast cell counts, sizes, and activation levels. Together, these data offer a different perspective on neuronal–mast cell signaling, suggesting sex differences in gut mucosal immunology and pointing toward the need to consider sex as a relevant variable when investigating mucosal neural‐immune signaling pathways.

## CONFLICT OF INTEREST

The authors declare that there is no conflict of interest.

## AUTHOR CONTRIBUTIONS

LAS conceptualized and designed the study, acquired data, and drafted and critically revised the manuscript. SAT conceptualized and designed the study and critically revised the manuscript.
